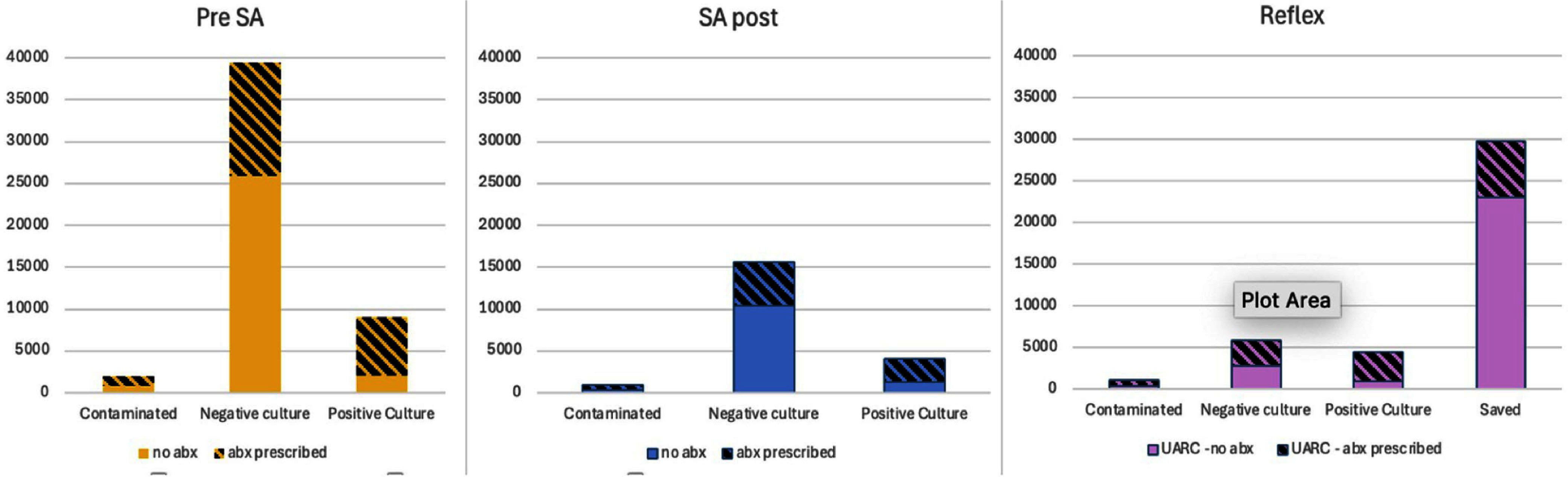# Impact of Urinalysis Reflex to Urine Culture Program on Antibiotic Use

**DOI:** 10.1017/ash.2025.320

**Published:** 2025-09-24

**Authors:** Adam Zimilover, Sammy Cheng, Erika Orner, Kelsie Cowman, Phyu Thwe, Wendy Szymczak, Inessa Gendlina

**Affiliations:** 1 Montefiore Medical Center - Albert Einstein College of Medicine; 2 Albert Einstein College of Medicine; 3 Montefiore Medical Center; 4 Einstein Montefiore

## Abstract

**Background:** Urinalysis (UA) with reflex to urine culture (UARC) protocols aim to optimize diagnostic testing and reduce unnecessary antibiotic use in hospitalized patients. By limiting urine cultures to cases where initial urinalysis results meet predefined criteria, UARC protocols can minimize false-positive results and reduce overtreatment. This study examines the impact of a UARC protocol implemented across a hospital system on urine culture volume and antibiotic utilization. **Methods:** A UARC protocol was implemented at our institution, performing urine cultures for UA specimens with ≥5 WBC/HPF. This study was an interrupted time-series analysis that compared the pre-implementation period (October 2022–June 2023) and the post-implementation period (January 2024–September 2024), with data elements abstracted from the electronic medical record. Antibiotic exposure within 48 hours before and 168 hours after urine specimen collection was evaluated. Comparisons were made using chi-square and Wilcoxon rank-sum tests, with p-values A total of 107,646 urine specimens were analyzed with 49,504 in the pre-implementation period and 58,142 post-implementation. Following UARC introduction, only 51.3% of reflex orders continued on to urine culture (29,836/58,142). Overall urine specimen orders resulting in antibiotic utilization decreased from 39.2% to 33.5% (p Implementing a UARC protocol significantly reduced urine culture volumes and antibiotic utilization, demonstrating its effectiveness in diagnostic and antimicrobial stewardship. While overall antibiotic use decreased, the unchanged treatment duration among recipients suggests complete courses were maintained with the reduction in culture orders serving as the mechanism driving this change. These findings support UARC protocols as valuable tools for reducing antibiotic use and optimizing healthcare resources. Further research should refine reflex criteria and assess long-term clinical outcomes.

**Figure:**
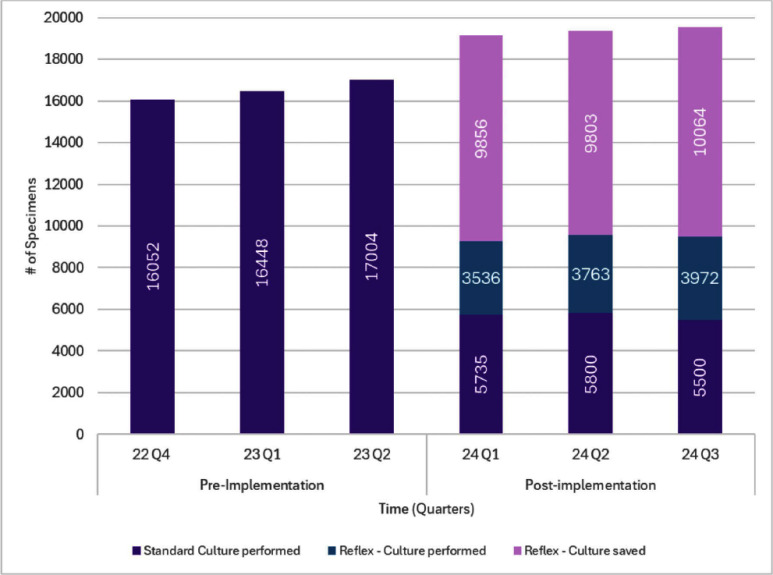

**Figure:**
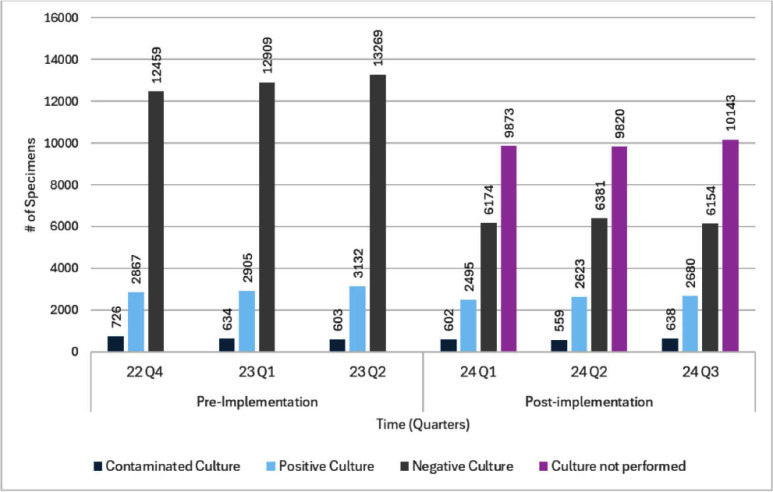

**Figure:**